# Increased N-Ethylmaleimide-Sensitive Factor Expression in Amygdala and Perirhinal Cortex during Habituation of Taste Neophobia

**DOI:** 10.1155/2016/2726745

**Published:** 2015-12-29

**Authors:** Beatriz Gómez-Chacón, Fernando Gámiz, Thomas C. Foster, Milagros Gallo

**Affiliations:** ^1^Department of Psychobiology, Center for Biomedical Research (CIBM), Institute of Neurosciences, University of Granada, Avenida del Conocimiento, s/n, 18071 Granada, Spain; ^2^Department of Neuroscience, McKnight Brain Institute, University of Florida, P.O. Box 100244, Gainesville, FL 32610-0244, USA

## Abstract

Interactions between GluR2 and N-ethylmaleimide-sensitive factor (NSF) mediate AMPA receptors trafficking. This might be linked with molecular mechanisms related with memory formation. Previous research has shown basolateral amygdala (BLA) dependent activity changes in the perirhinal cortex (PRh) during the formation of taste memory. In the present experiments we investigate both the behavioral performance and the expression profile of NSF and GluR2 genes in several brain areas, including PRh, BLA, and hippocampus. Twenty-one naïve male Wistar rats were exposed to a saccharin solution (0.4%) during the first (novel), the second (Familiar I), and the sixth presentation (Familiar II). Total RNA was extracted and gene expression was measured by quantitative PCR (qPCR) using TaqMan gene expression assays. In addition the expression of the synaptic plasticity related immediate early genes, Homer 1 and Narp, was also assessed. We have found increased expression of NSF gene in BLA and PRh in Group Familiar I in comparison with Familiar II. No changes in the expression of GluR2, Homer 1, and Narp genes were found. The results suggest the relevance of a potential network in the temporal lobe for taste recognition memory and open new possibilities for understanding the molecular mechanisms mediating the impact of sensory experience on brain circuit function.

## 1. Introduction

Taste neophobia refers to the reluctance to ingest novel tasting edibles. As long as the taste has no negative consequences, a learning process called habituation of neophobia takes place, leading to increased consumption when the taste is recognized as safe. Safe taste memory in the rat has been proposed as a model of recognition memory useful for studies of the molecular substrates of memory [1]. Thus, animal models of safe taste memory represent a privileged opportunity to study the impact of sensory experience on brain circuit function.

The formation of safe taste memories has been linked to protein synthesis in temporal lobe areas, including the perirhinal cortex (PRh) and hippocampus (HC) [2]. A relevant role of the glutamatergic transmission in the basolateral amygdala (BLA) has also been previously proposed. Thus, blocking NMDA receptors with MK-801 disrupts safe taste memory formation [3]. Moreover, we have previously reported that BLA lesions disrupt both the attenuation of taste neophobia and familiarity-related changes in PRh activity [4]. These results suggest the relevance of changes in synaptic efficacy in a temporal network, including BLA and PRh, for the acquisition and maintenance of safe taste memories. Postsynaptic trafficking of AMPA receptors plays a crucial role in regulating synaptic strength and memory [5–8]. Thus, the stabilization of long-term potentiation (LTP) and memories involves synaptic addition of GluR2 subunit-containing AMPA receptors (AMPARs) from the extrasynaptic pool. After LTP induction GluR2-lacking AMPARs are inserted in the synapses. The stabilization of LTP involves switching from GluR2-lacking AMPARs to GluR2-containing AMPARs. This process is mediated by interactions between GluR2 and N-ethylmaleimide factor (NSF) [9, 10]. Disrupting NSF/GluR2 interaction by inhibitory peptides in the lateral amygdala impaired long-term fear conditioned memory [11] and, in the dorsal hippocampus, interfered with long-term contextual fear memory and object-location recognition memory [12].

One of the mechanisms proposed for maintaining both LTP in the hippocampus [13–16] and a variety of memories [17–20] relies on an atypical protein kinase termed protein kinase Mzeta (PKM*ζ*). We have found that inhibition of PKM*ζ* by an inhibitory peptide (ZIP) in the BLA attenuates conditioned taste aversion suggesting interference with the formation of a safe taste memory [21]. Since it has been demonstrated that PKM*ζ* maintains hippocampal LTP [22] and amygdala-dependent fear memory [23] by regulating GluR2-dependent AMPARs trafficking, it could be proposed that NSF/GluR2 interactions in temporal areas might be involved in safe taste recognition memory.

In the present experiments we investigate both the behavioral performance and the expression profile of NSF and GluR2 genes in BLA, HC, and PRh after exposure to a saccharin solution during the first (novel), the second (Familiar I), and the sixth presentation (Familiar II). In addition expression of the synaptic plasticity related immediate early genes, Homer 1 and Narp, was also assessed.

## 2. Materials and Method

### 2.1. Animals

Twenty-one naïve male Wistar rats (7 weeks of age, mean: 275 g) were used. They were housed individually in standard hanging cages (44 × 30 × 20 cm) and maintained on a 12-hour light-dark cycle (lights on at 08:00 h). The humidity was kept at 55% and the temperature at 20–24°C. Rats were given food* ad libitum* and water until the experiment started when water access was restricted. Animals were randomly distributed in three experimental groups: (1) rats sacrificed after the initial experience drinking the sodium saccharin solution on day 1 (novel group, *n* = 7); (2) rats sacrificed after drinking the familiar taste solution on day 2 (Familiar I, *n* = 7); (3) a group of rats sacrificed after drinking the familiar taste solution on day 6 (Familiar II, *n* = 7) (Table 1). Only the consumption of the Familiar II groups was taken into account for the behavioral analysis.

### 2.2. Behavioral Procedure

Behavioral testing took place in the home cages. During the acclimation to the deprivation schedule, water intake was recorded for nine days during the morning 20-minute drinking period. Once the water intake baseline (BL) was stabilized, the rats received access to a 0.4% sodium saccharin solution during the next six daily drinking sessions. The rats were sacrificed 30 minutes after the drinking period at different days depending on the group they were assigned, that is, the first day (novel), the second day (Familiar I), and the sixth day (Familiar II) (Table 1). All the procedures were approved by the University of Granada Ethics Committee for Animal Research and were in accordance with the European Communities Council Directive 86/609/EEC.

### 2.3. Histology and Sample Preparation

Following the behavioral testing, animals from each group were anesthetized with isoflurane and sacrificed by decapitation. The brain was removed quickly and the PRh, HC, and BLA were dissected and immediately frozen in liquid nitrogen. The tissues were stored at −80°C until used.

Total RNA was extracted from samples by homogenization using the RNeasy Lipid Tissue Mini Kit (Qiagen), according to the manufacturer's protocols. Total cDNA was performed using High-Capacity cDNA Reverse Transcription Kits (Applied Biosystems, USA). Reverse transcription was performed using 200 ng of total RNA from each sample. A solution-phase assay was carried out in 96- and 384-well microplates (Applied Biosystems).

### 2.4. TaqMan OpenArray Real-Time PCR

Gene expression was measured by quantitative PCR (qPCR) using TaqMan gene expression assays. OpenArray Real-Time PCR plate format 18(3x) × 48 was used. The gene expression assays included GluR2/Gria2 (glutamate receptor 2) [Rn00568514_m1], HOMER 1 (homer protein homolog 1) [Rn00581785_m1], Narp/NPTX 2 (neuronal pentraxin-2), and NSF (N-ethylmaleimide-sensitive) [Rn00572694_m1]. GADPH (glyceraldehyde-3-phosphate dehydrogenase) [Rn01775763_g1] and ACTB (Actin, beta) [Rn00667869_m1] were used as endogenous controls. The OpenArray AccuFill system was used for loading the sample into OpenArray plates. The samples were analyzed by Real-Time quantitative PCR (RT-qPCR) using TaqMan Gene Expression assays and OpenArrayTM NT Cycler (Applied Biosystems). PCR products are measured as the fluorescence signal after each cycle with the OpenArray Real-Time qPCR Analysis Software (Applied Biosystems, version 1.0.4). The Delta-Delta Comparative Threshold (ΔΔCt) method was used to quantify the fold change between the samples [24]. The threshold-cycle (Ct) value of each target gene was normalized by subtraction of the Ct value from average of two housekeeping genes (beta-actin and GAPDH) as internal control (ΔCt = Ct target – Ct control genes). It was further normalized with the control group for obtaining the fold change (RQ). Reactions that have high Ct values (>35) were cut off and the threshold amplification curve was adjusted to 2.0.

### 2.5. Data Analyses

Repeated measures analyses of variance (ANOVAs) were used to analyze the consumption along the drinking sessions for animals that completed all sessions (i.e., Familiar II group). One way ANOVAs were performed to compare consumption of the different groups. Expression data analyses were performed using DataAssist software (Applied Biosystems, version 3.01). Relative Quantification (RQ) values (relative levels of RNA expression) were calculated using the comparative Ct method with endogenous controls to normalize the data. Extreme values ranging more than two standard deviations were removed from the sample as that might create artificial baseline levels of gene expression. Before analysis, the data were tested for distribution and found to be normally distributed. Repeated measures analyses of variance (ANOVAs) were used to compare each gene expression in each brain zone.* Post hoc* Fisher LSD test comparisons between the groups were used. Differences were considered as statistically significant at *p* < 0.05.

## 3. Results

### 3.1. Taste Memory

**Figure 1:**
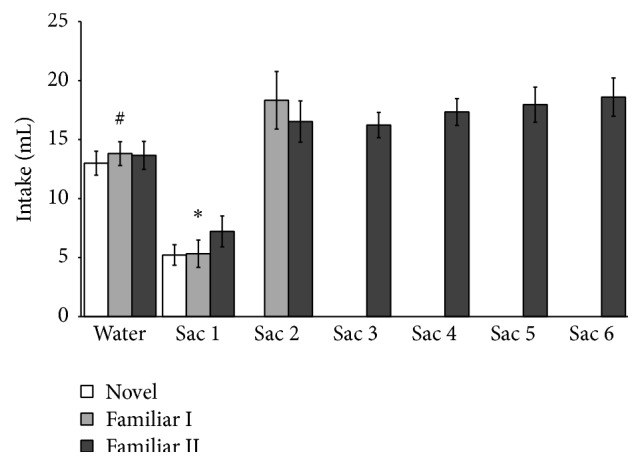
Mean (± SEM) intake during the last day of water baseline (BL) and the six saccharin solution (Sac) exposure sessions. For clarity all the groups are included in the figure but the statistical values correspond to group Familiar II (*∗* versus Sac 2, 3, 4, 5, and 6, *p* < 0.05; # versus Sac 1, *p* < 0.05).

Figure 1 shows mean (± SEM) consumption of water during the last baseline session and saccharin solution during the sixth exposure sessions. As mentioned above the statistical analyses across all sessions are based on the data of Familiar II groups since they were sacrificed after the end of the six daily saccharin solution drinking sessions. ANOVA for individual days indicated that the groups did not differ in water intake during the last baseline day (*F*(2,18) = 0.18; *p* > 0.05) or in saccharin consumption on days 1 (*F*(2,18) = 0.99, *p* > 0.05) and 2 (*F*(1,12) = 0.36; *p* > 0.05). Mean (± SEM) saccharin intake by all the groups is shown in Table 1.

The neophobic response to the saccharin solution was evident as a significant (*F*(1,6) = 9.82, *p* < 0.05) decreased intake of saccharin solution was found on day 1 in comparison with the last baseline. A repeated measures ANOVA, performed on data from rats in Familiar II group (days 1–6), found a significant main effect of days, *F*(5, 30) = 9.73, *p* < 0.001.* Post hoc* comparisons by Fisher LSD test revealed that intake on day 1 was significantly lower than on days 2, 3, 4, 5, and 6 (*p*s > 0.05), indicating the attenuation of neophobia.

### 3.2. Taste Memory-Related Gene Expression

**Figure 2:**
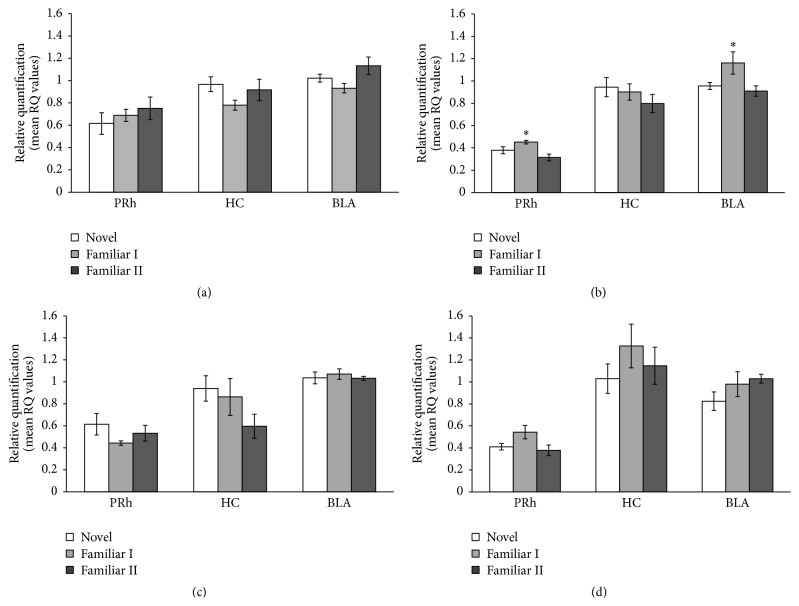
Fold change values for the genes GluR2 (a), NSF (b), Homer 1 (c), and Narp (d) in PRh, HC, and BLA of novel, Familiar I, and Familiar II groups. *∗* versus Familiar II group (*p* < 0.05).

Figure 2 shows the fold change values for the genes GluR2 (Figure 2(a)), NSF (Figure 2(b)), Homer 1 (Figure 2(c)), and Narp (Figure 2(d)) in PRh, HC, and BLA. Repeated measure ANOVAs revealed significant main effect of taste familiarity in the expression of NSF in PRh (*F*(2,14) = 7.34; *p* < 0.05) and BLA (*F*(2,14) = 3.81; *p* < 0.05). Fisher* post hoc* analyses yielded significant upregulation after the second taste exposure (Familiar I) compared with the sixth exposure (Familiar Il) (*p* < 0.05). No significant differences were found in HC. Likewise, there were no significant differences in any brain area regarding GluR2, Homer 1, and Narp (*p*s > 0.05).

## 4. Discussion 

It has been previously reported that NSF/GluR2 interaction in the dorsal hippocampus is required for a type of visual recognition memory including object-location information [12]. To the best of our knowledge in the present study we show for the first time changes of NSF expression in BLA and PRh related with taste recognition memory. NSF expression in both areas is upregulated when a safe taste becomes familiar after the second presentation in comparison with a later phase after six taste exposure sessions leading to a long-term memory trace.

In accordance with a definition of the neophobic response to a novel taste, taking into account not only decreased consumption during the first encounter but also later increases upon subsequent exposure sessions [25], our behavioral results confirm neophobia to the saccharin solution since the rats drank a lower amount during the first exposure than during the previous water session and the subsequent saccharin presentation. Thus, attenuation of taste neophobia required only one exposure session because there were no differences between the amounts drank along the subsequent five presentations. This is consistent with previous reports that applied a similar sodium saccharin concentration and number of taste exposure sessions [26]. The added sessions may have allowed long-term formation of taste memory.

Regarding the gene expression profiles the main finding merits discussion. NSF expression significantly increased during the second in comparison with the sixth taste presentation. Such an increase cannot be attributed to overall motor, sensory, or motivational effects associated with drinking the taste solution since there were no intake differences between the second and sixth drinking session. The fact that the significant increase in NSF expression takes place by the second taste exposure can have two different interpretations. First, it could be proposed that NSF/GluR2 interaction was necessary selectively during consolidation of the taste memory trace. This interpretation is consistent with the results reported by Joels and Lamprecht [11] showing that NSF/GluR2 interaction was required for fear memory consolidation but not acquisition, retrieval, or maintenance. However, given the fact that the memory consolidation hypothesis has been recently questioned [27], a second interpretation in terms of a selective role of NSF/GluR2 in short-term but not long-term habituation seems to be more feasible. According to Wagner's “Sometimes Opponent Processes” (SOP) theory [28] the mechanisms involved in short-term habituation can be independent of those leading to long-term habituation. Thus, a role of NSF in short-term but not long-term habituation is conceivable since NSF expression decreases significantly by the sixth exposure in spite of the maintenance of the taste memory. This has been demonstrated also using spatial memory tasks with other AMPA receptor subunits which are relevant for short-term but not long-term memory. The GluA1 AMPA receptor subunit knockout mouse exhibits selective impairment performing working memory tasks that involve short-term habituation but not in reference to long-term memory tasks [29].

The selective regional distribution of the increased NSF expression in BLA and PRh, but not HC, supports the relevance of an amygdalar-perirhinal network in the formation of safe taste memories. Whilst the anatomical circuits that subserve the formation of aversive taste memories have been extensively investigated, especially the interaction between the insular cortex and the amygdala in the acquisition of conditioned taste aversion [30, 31], the scarce data on brain areas involved in the attenuation of taste neophobia point to a crucial role of a network formed by BLA and PRh [4]. Extensive anatomical and electrophysiological evidence indicates reciprocal functional connections of the PRh, BLA, and HC among other taste related areas. This might be the substrate underlying its safe taste memory formation [32]. The fact that no changes of NSF expression in HC have been found in the present study was expected. Although protein synthesis in the dorsal hippocampus has been reported to be involved in the formation of safe taste memories [2], we have previously found no changes in dorsal hippocampus c-fos expression during attenuation of taste neophobia [4]. In turn, there is ample evidence supporting a selective hippocampal role in visual recognition memory in tasks that require the animal to remember the spatial location of the objects [33]. Accordingly, disruption of NSF/GluR2 interaction in dorsal hippocampus by infusing the interference peptide pep2m impaired maintenance of object-location recognition memory [12].

Since the proposed action mechanism of NSF for regulating AMPA trafficking lies in binding the AMPA receptor subunit GluR2 thus stabilizing postsynaptic transmission, the absence of changes in the pattern of GluR2 expression found in our study can be explained by the fact that this process is thought to involve mobilization of GluR2 subunits from extrasynaptic pools not requiring synthesis* de novo* during the temporal window (30 min) examined [5, 10]. Also the lack of changes in the expression of the immediate early genes Homer 1 and Narp does not allow us to discard a potential involvement in taste memory formation unnoticed due to regional/temporal differences in consolidation. While Homer 1 has been related with glutamatergic neurotransmission in the gustatory cortex [31], a modest increase of Narp staining in the dentate gyrus has been found during object-location recognition memory [34]. However, no previous work has reported a specific relationship between expression changes of these immediate early genes and taste memory. Together, the results suggest that, at least for the regions examined, Homer 1 and Narp may not be involved in taste memory. Therefore, our data are consistent with the lack of results on this issue and prompt further research on the molecular basis of safe taste memory.

In all, our results suggest a role for NSF in short-term habituation of the neophobic response which can be connected with the proposed role of PKM*ζ* on maintaining LTP [13–16] and memory [17–20, 35]. PKM*ζ* role in memory seems to be connected with the regulation of GluR2-dependent AMPARs trafficking [22, 23]. Our results showing attenuation of conditioned taste aversion by ZIP [21] and increase in NSF expression during formation of the safe taste memory add to previous data to link both mechanisms in the BLA. Furthermore, a similar pattern of NSF expression in PRh breaks new ground for research on the brain mechanisms of recognition memory.

## Figures and Tables

**Table 1:** Timeline depicting the experimental procedure. Mean (± SEM) intake during the six saccharin solution (Sac) exposure sessions (*n* = number of animals per group; Sac = 0.4% saccharin solution; ^†^sacrifice 30 min after the drinking period).

Groups	Days					
	1	2	3	4	5	6
Novel	Sac^†^ *n* = 7					
	5.21 ± 0.86					

Familiar I	Sac	Sac^†^ *n* = 7				
	5.32 ± 1.15	18.33 ± 2.44				

Familiar II	Sac	Sac	Sac	Sac	Sac	Sac^†^ *n* = 7
	7.21 ± 1.30	16.53 ± 1.75	16.23 ± 1.07	17.34 ± 1.13	17.96 ± 1.48	18.60 ± 1.62
